# Supplementary material for: Immune-enhancing neutrophils reprogrammed by subclinical low-dose endotoxin in cancer treatment
Source: EMBO Mol Med. 2024 Jul 15;16(8):1886–900. doi: 10.1038/s44321-024-00100-7 (PMC11319772; doi:10.1038/s44321-024-00100-7)
Supplement: Supplementary file 1 — Appendix [file 44321_2024_100_MOESM1_ESM.pdf]

Appendix

Immune-enhancing neutrophils reprogrammed by subclinical low-dose endotoxin conducive for cancer treatment

Yao Zhang, Christina Lee, Shuo Geng, Jing Wang, Udipta Bohara, Jacqueline Hou, Ziyue Yi, Liwu Li*

Department of Biological Sciences, Virginia Tech, Blacksburg, VA 24061-0910

Table of Contents

Appendix Figure S1……………………………………………………………………………………1

Appendix Figure S2……………………………………………………………………………………2

Appendix Figure S3……………………………………………………………………………………3

Appendix Figure S4……………………………………………………………………………………4

Appendix Figure S5……………………………………………………………………………………5

Appendix Figure S6……………………………………………………………………………………6

Appendix Figure S7……………………………………………………………………………………7

Appendix Figure S8……………………………………………………………………………………8

Appendix Figure S9……………………………………………………………………………………9

Appendix Figure S10…………………………………………………………………………………10

Appendix Table S1. Reagents and tools……………………………………………………………...11

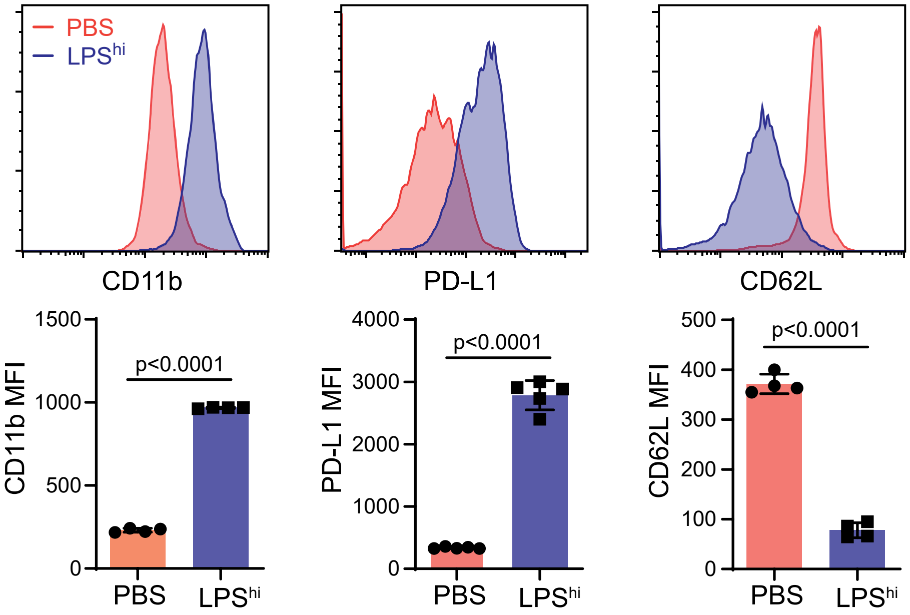

Appendix Figure S1. Polarization of neutrophils to an immune-supressive phenotype by high dose LPS.

Bone marrow neutrophils were treated with a high dose of LPS (100ng/ml) or PBS as a control overnight, then subjected to flow cytometry. CD11b, PD-L1, and CD62L expression on CD11b +Ly6G+ neutrophils were analyzed. Representative plots and quantification results are shown. n≥4. Significance was calculated by unpaired two-tailed student *t* test, error bars represent means ±SD.

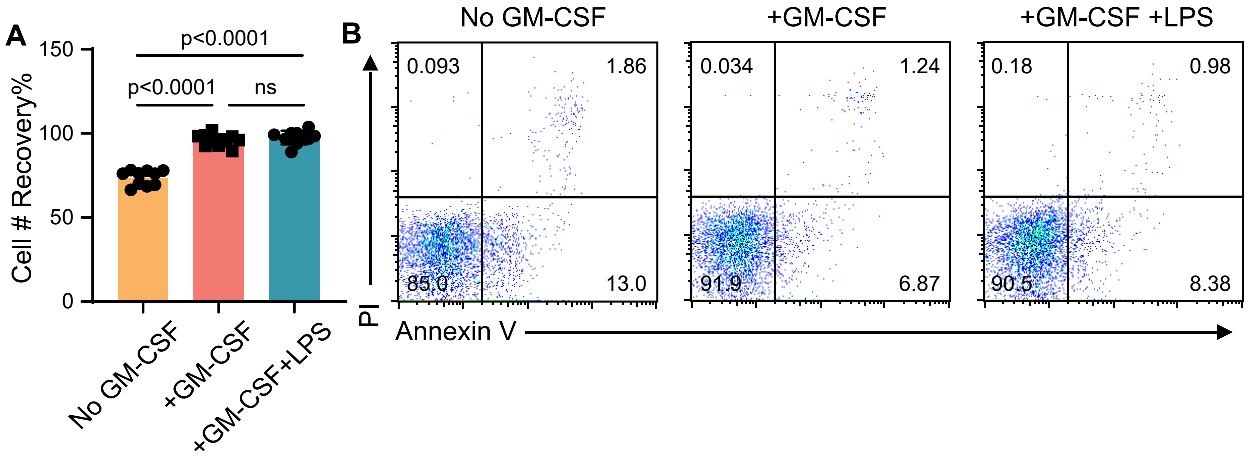

Appendix Figure S2. Neutrophil survival effectively sustained by low-dose GM-CSF.
A. Bone marrow neutrophils were cultured in the RPMI complete medium with or without GM-CSF (1ng/ml), treated with or without LPS (100pg/ml) overnight, then subjected to cell counting. n=10. Significance was calculated by unpaired two-tailed student *t* test, error bars represent means ± SD.
B. Representative flow cytometry dot plots of annexin V staining, pre-gated on CD11b+Ly6G+ neutrophils.

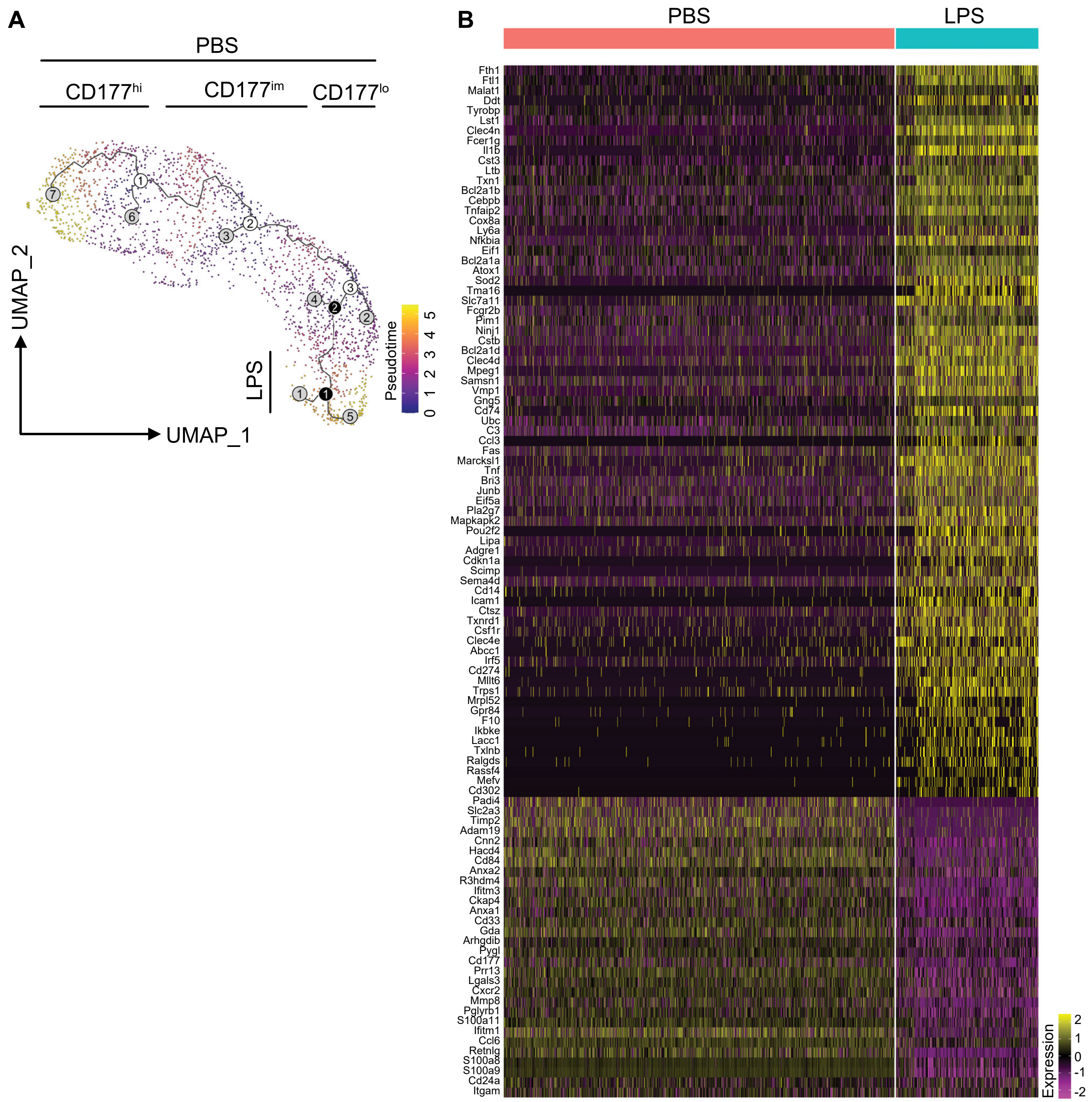

Appendix Figure S3. Pseudo time trajectory analysis of single cell RNAseq data.
A. Trajectory analysis on a pseudo time map of various neutrophil clusters, with color schemes reflecting the pseudo time origin and destination. **B.** Heatmaps of representative genes in purified neutrophils challenged with super-low dose of LPS (100pg/ml).

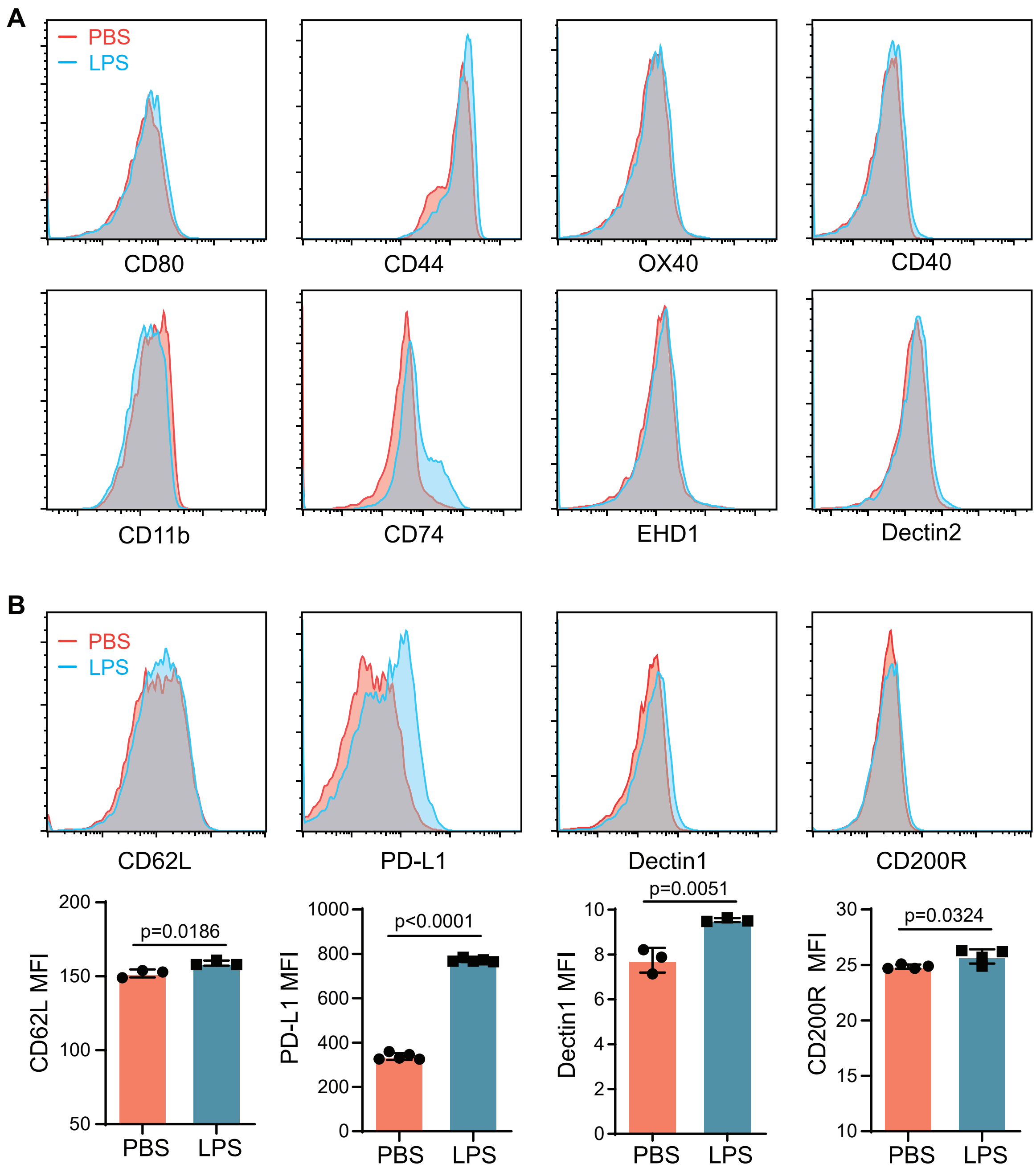

Appendix Figure S4. Expressions of surface molecules on neutrophils trained by low dose of LPS.

Bone marrow neutrophils were treated with super-low dose of LPS (100pg/ml) or PBS as control overnight, then subjected to flow cytometry analysis. **A.** Representative stacked histogram plots of selected proteins in CD11b+Ly6G+ neutrophils. Quantitative analysis of mean fluorescence intensity (MFI) is shown in Figure 1A. **B.** Additional surface molecules including CD62L, PD-L1, Dectin1, and CD200R expression on CD11b+Ly6G+ neutrophils were analyzed. Representative plots and quantification results are shown. n≥3. Data are represented as means ± SD. Significance was calculated by unpaired two-tailed student *t* test.

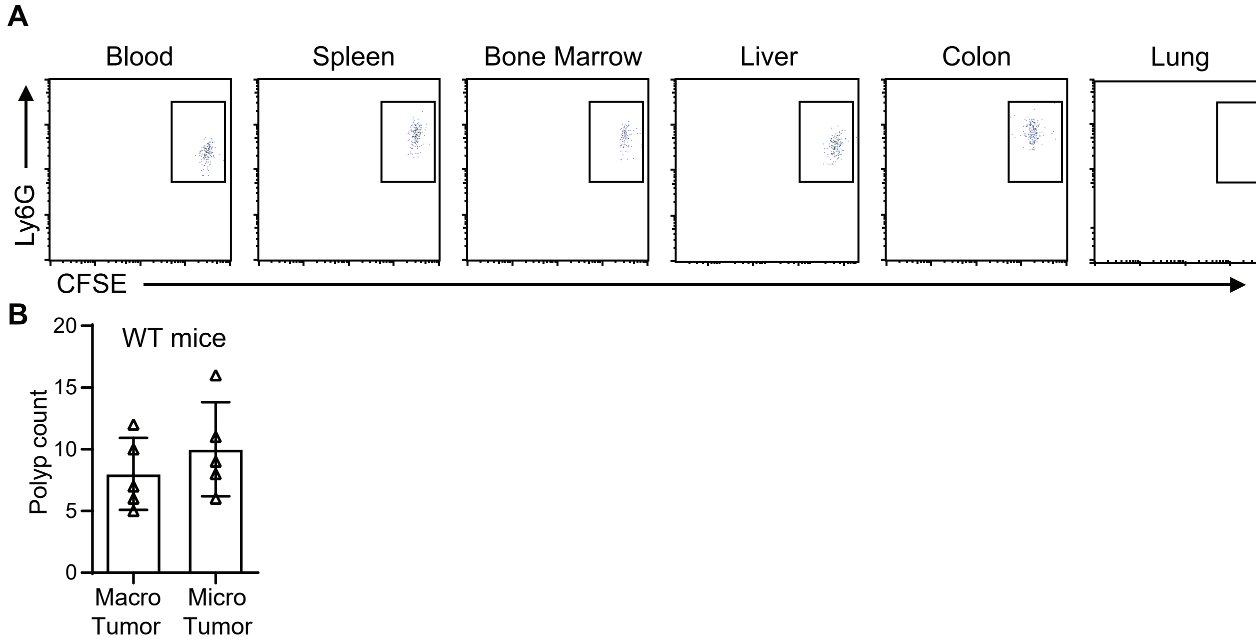

Appendix Figure S5. In vivo tracking of adoptively transferred neutrophils.
A. Representative flow cytometry dot plots of adoptively transferred CFSE-labeled neutrophils, harvested from recipient mice 24 hours after the transfer. CFSE positive cells were gated on single live Ly6G+CD11b+ cells.
B. WT mice were under AOM -DSS treatment as described in the Methods section without any neutrophil transfusion. At the end of the AOM-DSS regimen, tumor load was counted. n=5.

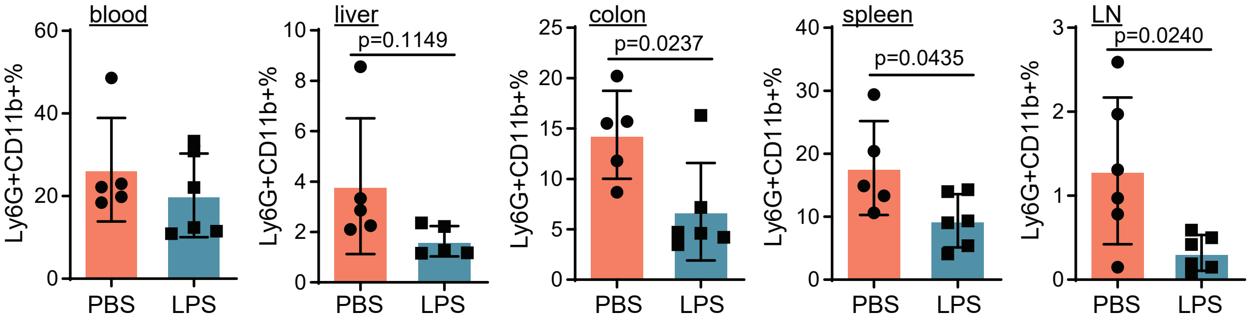

Appendix Figure S6. Reduction of tissue inflammation by transfusion with LPS-primed neutrophils. The percentages of neutrophils in various tissues from AOM-DSS challenged mice with adoptive transfer of neutrophils were analyzed by flow cytometry. n≥5 per group. Data are represented as means ± SD. Significance was calculated by unpaired two-tailed student *t* test.

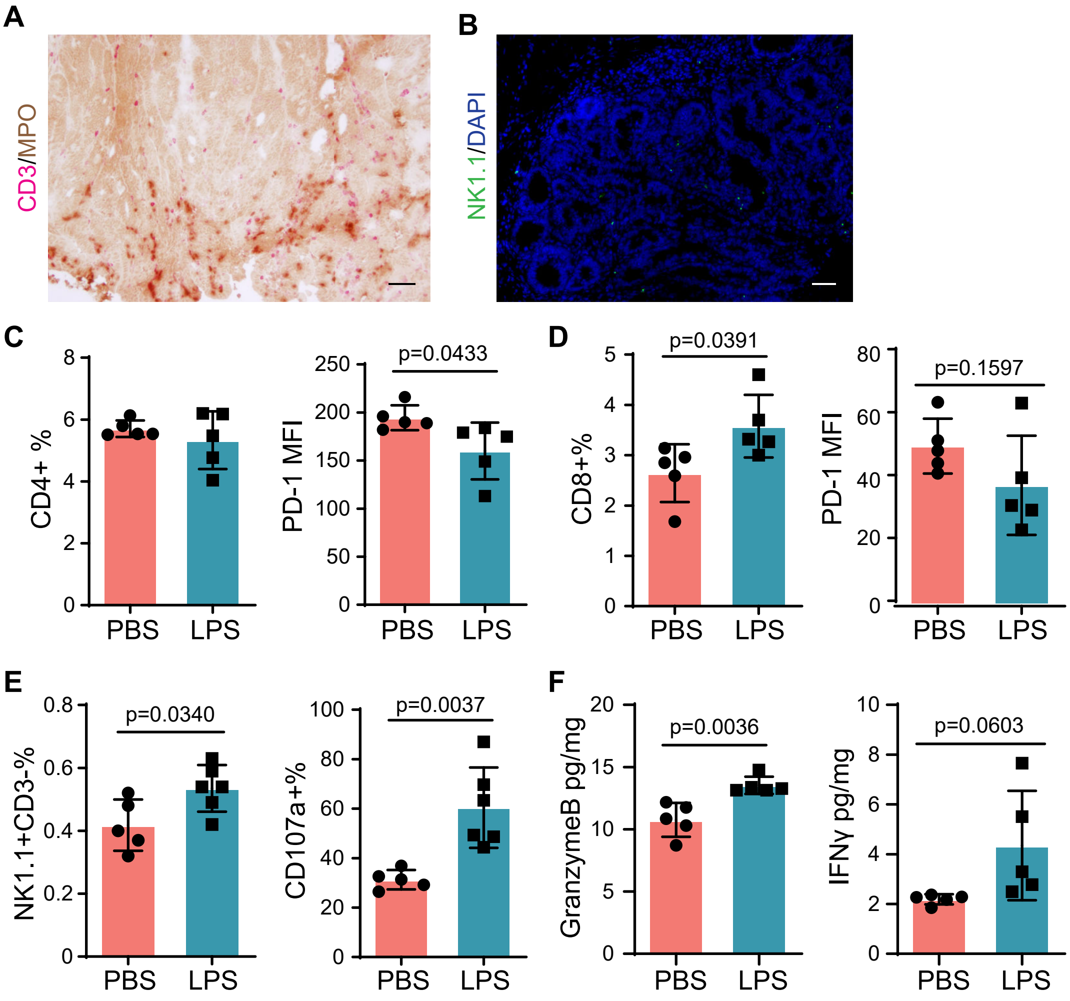

Appendix Figure S7. Enhanced anti-tumor environment in the colon by transfusion with LPS-primed neutrophils.

A. Representative image of immunohistochemistry staining of myeloperoxidase (MPO) and CD3 in colon tissue from AOM-DSS challenged mice with adoptive transfer of neutrophils. Scale bar: 50μm.

B. Representative image of immunofluorescence analysis of NK1.1 in colon tissue. Nuclei were stained with DAPI. Scale bar: 50μm.

C-F. Flow cytometry was performed on the colon lamina propria from AOM-DSS challenged mice with adoptive transfer of neutrophils, pre-gated in CD45+ cells. n≥5 per group.

C. The percentages of CD4+ T cells in the colon and PD-1 expression on CD4+ T cells. **D.** The percentages of CD8+ T cells in colon and PD-1 expression on CD8+ T cells. **E.** The percentages of NK cells in colon and CD107a expression on NK cells. **F.** Granzyme B and IFNγ levels in colon lysate, normalized on protein weight. n≥5. Data are represented as means ± SD. Significance was calculated by unpaired two-tailed student *t* test.

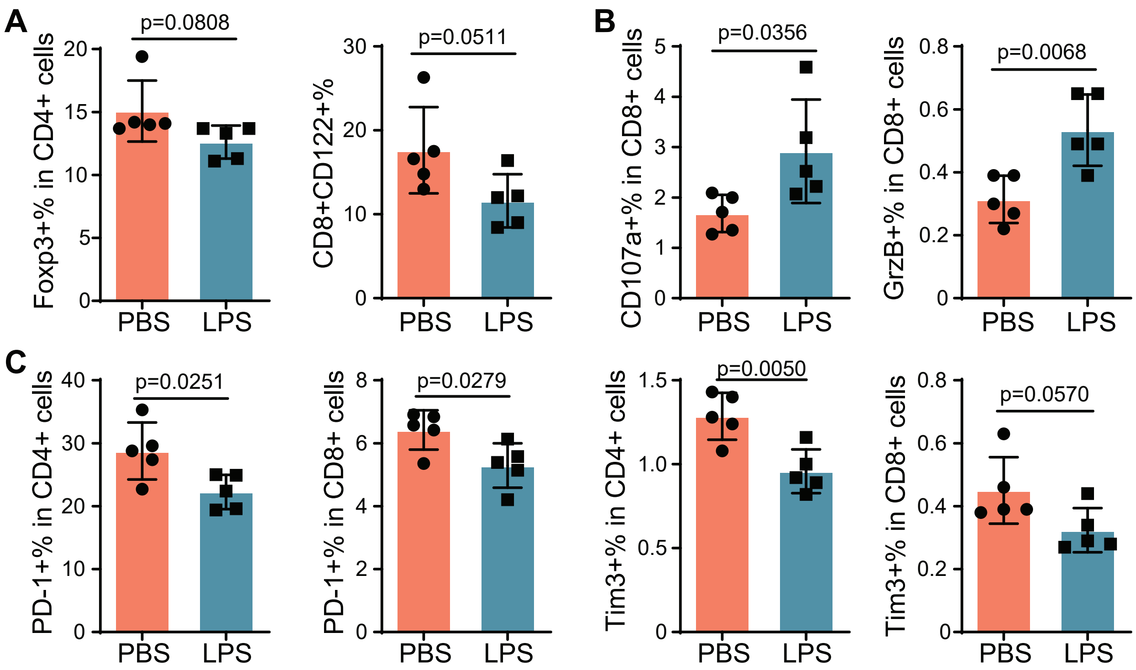

Appendix Figure S8. Enhanced T cell activities in the mesenteric lymph nodes by transfusion with LPS- primed neutrophils.

T cells in the mesenteric lymph nodes from AOM-DSS challenged mice with adoptive transfer of neutrophils were analyzed by flow cytometry. n≥5 per group. **A.** Foxp3 expression in CD4+ T cells and CD122 expression in CD8+ T cells. **B.** CD107a and Granzyme B (GrzB) levels in CD8 + T cells. **C.** PD-1 and Tim3 expression in T cells. n≥5. Data are represented as means ± SD. Significance was calculated by unpaired two-tailed student *t* test.

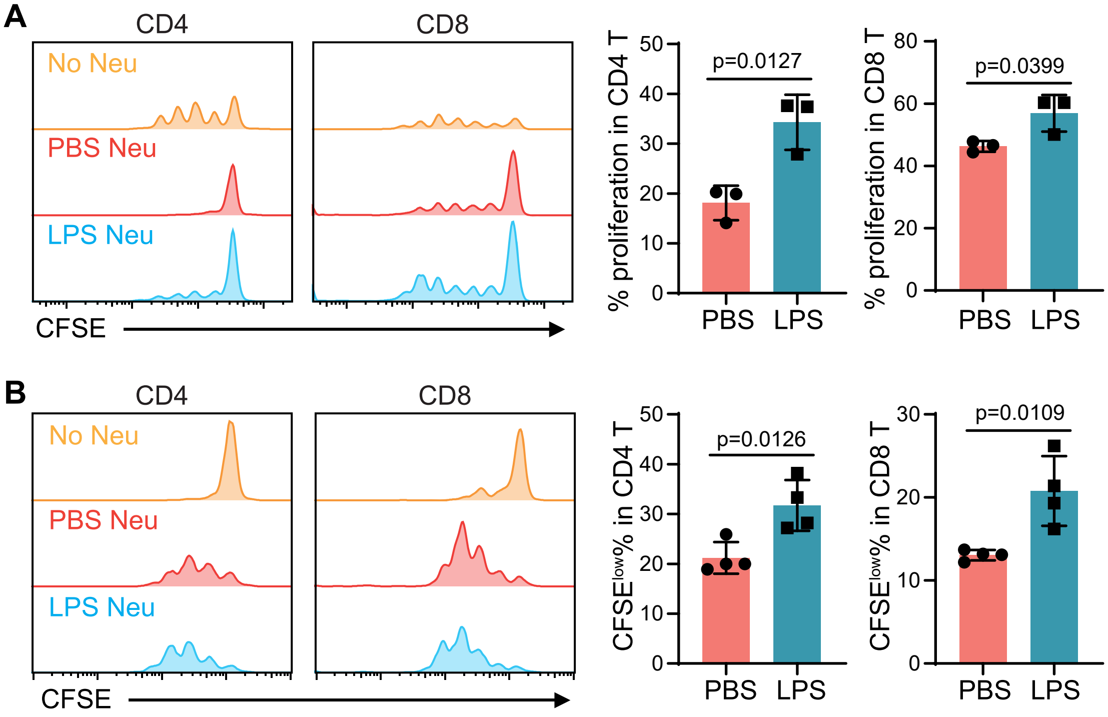

Appendix Figure S9. Enhanced T cell proliferation by LPS-primed neutrophils.
A. CFSE-labeled murine T cells were co- cultured with primed syngeneic neutrophils. Representative plot and quantification results of CFSE signal gated on CD4+ or CD8+ T cells. n=3.
B. CFSE-labeled human T cells were co-cultured with primed syngeneic neutrophils. Representative plot and quantification results of CFSE signal gated on CD4+ or CD8+ T cells. n=4. Data are represented as means ± SD. Significance was calculated by unpaired two-tailed student *t* test.

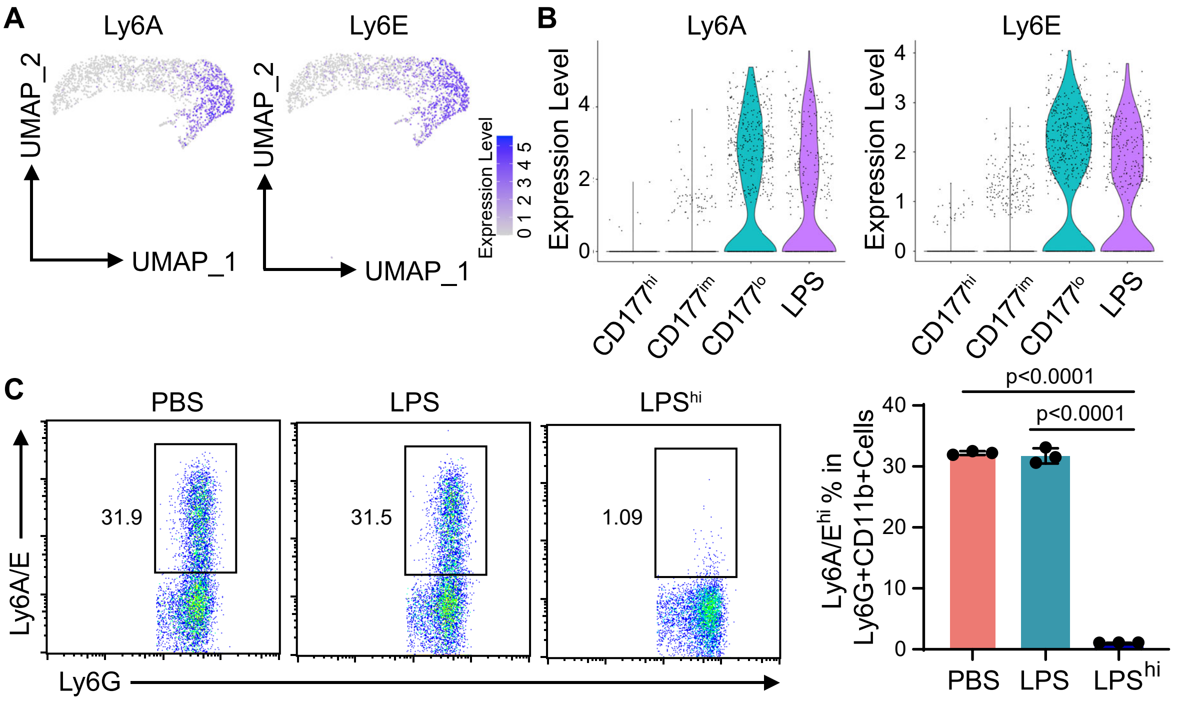

Appendix Figure S10. Appendix Figure S10. Expression of Ly6A/E in the different clusters of neutrophils.

A. Feature UMAP plot denoting the expression levels of Ly6A/E within various neutrophil subsets. **B.** Violin plots of gene expression distribution in different clusters of neutrophils. **C.** Representative flow cytometry plots and quantification of Ly6A/E expression levels gated on Ly6G+CD11b+ neutrophils. n=3. Data were represented as means ± SD. Significance was calculated by unpaired two-tailed student *t* test.

Appendix Table S1 List of antibodies used

Flow cytometry antibodies			
**Antibody**	**Supplier**	**Catalog number**	**Clone**
CD80	Biolegend	104708	16-10A1
CD40	Biolegend	124621	3/23
OX40	Biolegend	119413	ox-86
CD44	Biolegend	103029	IM7
CD74	Biolegend	151003	In1/CD74
Dectin1	Biolegend	144305	RH1
Dectin2	R&D Systems	FAB1525A	Q9JKF4
CD11b	Biolegend	101226	M1/70
CD4	Biolegend	100414	GK1.5
CD8	Biolegend	100712	53-6.7
PD-L1	Biolegend	124313	B7-H1
CD62L	Biolegend	104428	MEL-14
Ly6G	Biolegend	127606	1A8
Ki67	Biolegend	652407	16A8
PD-1	Biolegend	135206	29F.1A12
Tim3	Biolegend	119705	RMT3-23
granzymeB	Biolegend	515405	GB11
IFNγ	Biolegend	505808	XMG1.2
CD107a	Biolegend	121609	1D4B
CD122	Biolegend	123207	TM-β1
CD200R	Biolegend	123908	OX-110
CD69	Biolegend	104525	H1.2F3
NKG2A	Biolegend	142807	16A11
Foxp3	Biolegend	126403	MF-14
CD4	Biolegend	300518	RPA-T4
CD8	Biolegend	301014	RPA-T8
Ly6A/E	Biolegend	108111	D7
EHD1	Abcam	ab109747	EPR4955
Goat anti-Rabbit IgG (H+L) Alexa Fluor Plus 647	ThermoFisher	A32733	
Rabbit IgG Isotype Control	ThermoFisher	10500C	

Immunoblotting and immunofluorescence staining antibodies		
**Antibody**	**Supplier**	**Catalog number**
IRAK-M	Abcam	ab8116
pSTAT5	Cell Signaling	9359
STAT5	Cell Signaling	94205
PSMD10	Cell Signaling	12985
KLF4	Abcam	ab129473
β-actin	Santa Cruz	sc-47778 HRP
Anti-Rabbit IgG, HRP-linked	Cell Signaling	7074
Ki67	Cell Signaling	12202
beta-cantinen (active)	Cell Signaling	8814
NK1.1/CD161 (E6Y9G) Rabbit mAb (Alexa Fluor® 488 Conjugate)	Cell Signaling	92556S
Alexa Fluor® 594 Streptavidin	Biolegend	405240
Goat Anti-Rabbit IgG Antibody (H+L)	Vectorlabs	BP-9100-50
IRAK-M for IP	Abcam	ab307708
STAT1	Cell Signaling	9172
STAT3	Cell Signaling	12640
Lamin B1	Cell Signaling	13435
GAPDH	Cell Signaling	2118
Histone H3	Abcam	ab176842
CD3	Abcam	ab11089
MPO	Abcam	ab9593
