# Supplementary figures and images for: Immune-enhancing neutrophils reprogrammed by subclinical low-dose endotoxin in cancer treatment
Source: EMBO Mol Med. 2024 Jul 15;16(8):1886–900. doi: 10.1038/s44321-024-00100-7 (PMC11319772; doi:10.1038/s44321-024-00100-7)

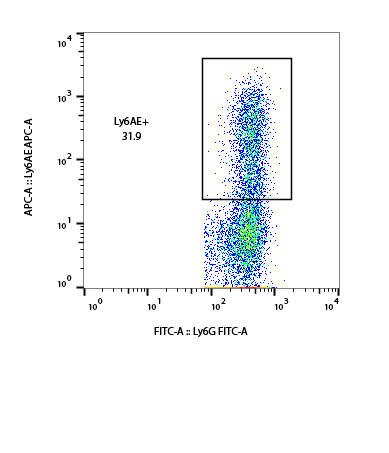

Supplement: Supplementary file 3 — Appendix Figure Source Data [file 44321_2024_100_MOESM3_ESM.zip › Appendix Figures/S10/1.jpg]

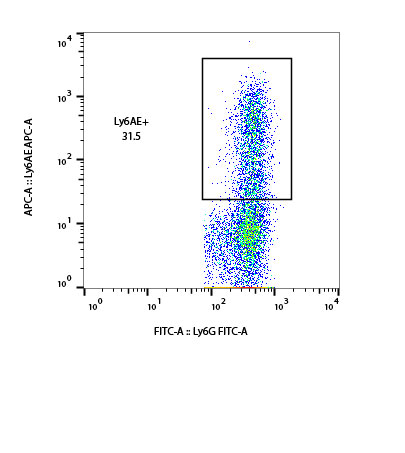

Supplement: Supplementary file 3 — Appendix Figure Source Data [file 44321_2024_100_MOESM3_ESM.zip › Appendix Figures/S10/2.jpg]

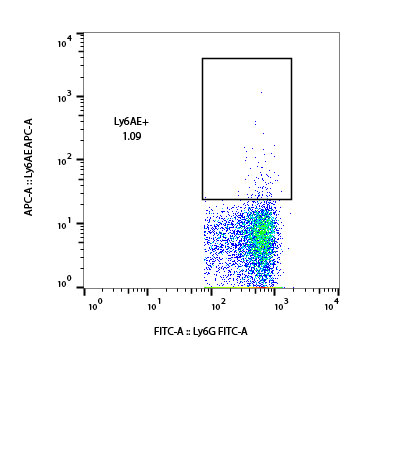

Supplement: Supplementary file 3 — Appendix Figure Source Data [file 44321_2024_100_MOESM3_ESM.zip › Appendix Figures/S10/3.jpg]

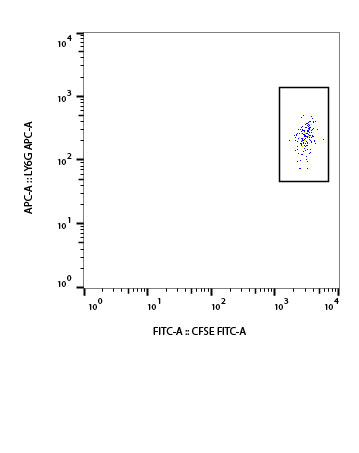

Supplement: Supplementary file 3 — Appendix Figure Source Data [file 44321_2024_100_MOESM3_ESM.zip › Appendix Figures/S5/blood.jpg]

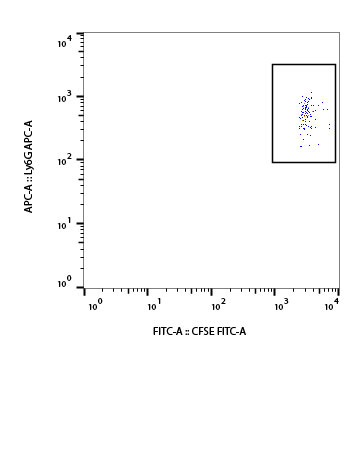

Supplement: Supplementary file 3 — Appendix Figure Source Data [file 44321_2024_100_MOESM3_ESM.zip › Appendix Figures/S5/Bone marrow.jpg]

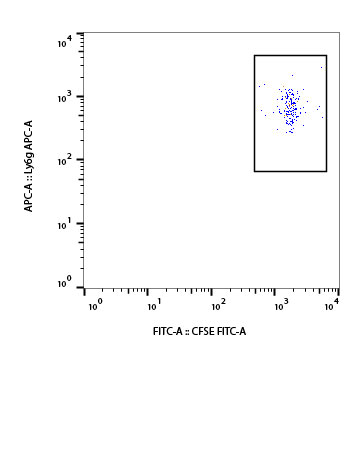

Supplement: Supplementary file 3 — Appendix Figure Source Data [file 44321_2024_100_MOESM3_ESM.zip › Appendix Figures/S5/colon.jpg]

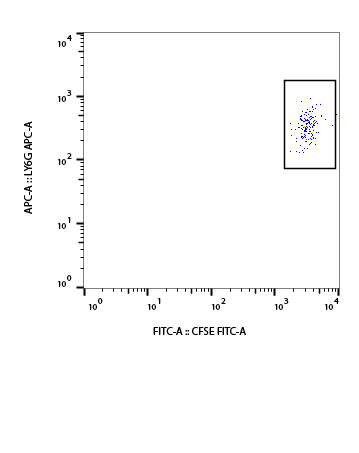

Supplement: Supplementary file 3 — Appendix Figure Source Data [file 44321_2024_100_MOESM3_ESM.zip › Appendix Figures/S5/liver.jpg]

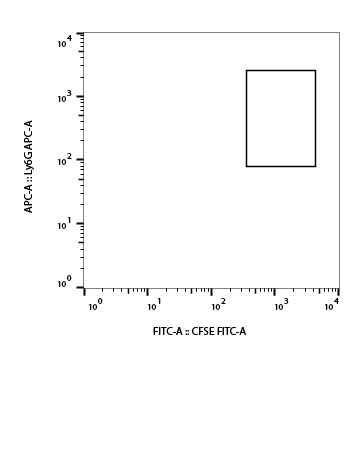

Supplement: Supplementary file 3 — Appendix Figure Source Data [file 44321_2024_100_MOESM3_ESM.zip › Appendix Figures/S5/Lung.jpg]

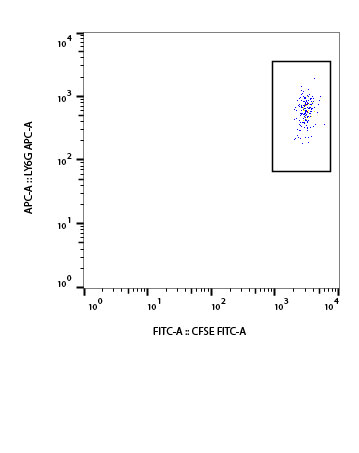

Supplement: Supplementary file 3 — Appendix Figure Source Data [file 44321_2024_100_MOESM3_ESM.zip › Appendix Figures/S5/spleen.jpg]

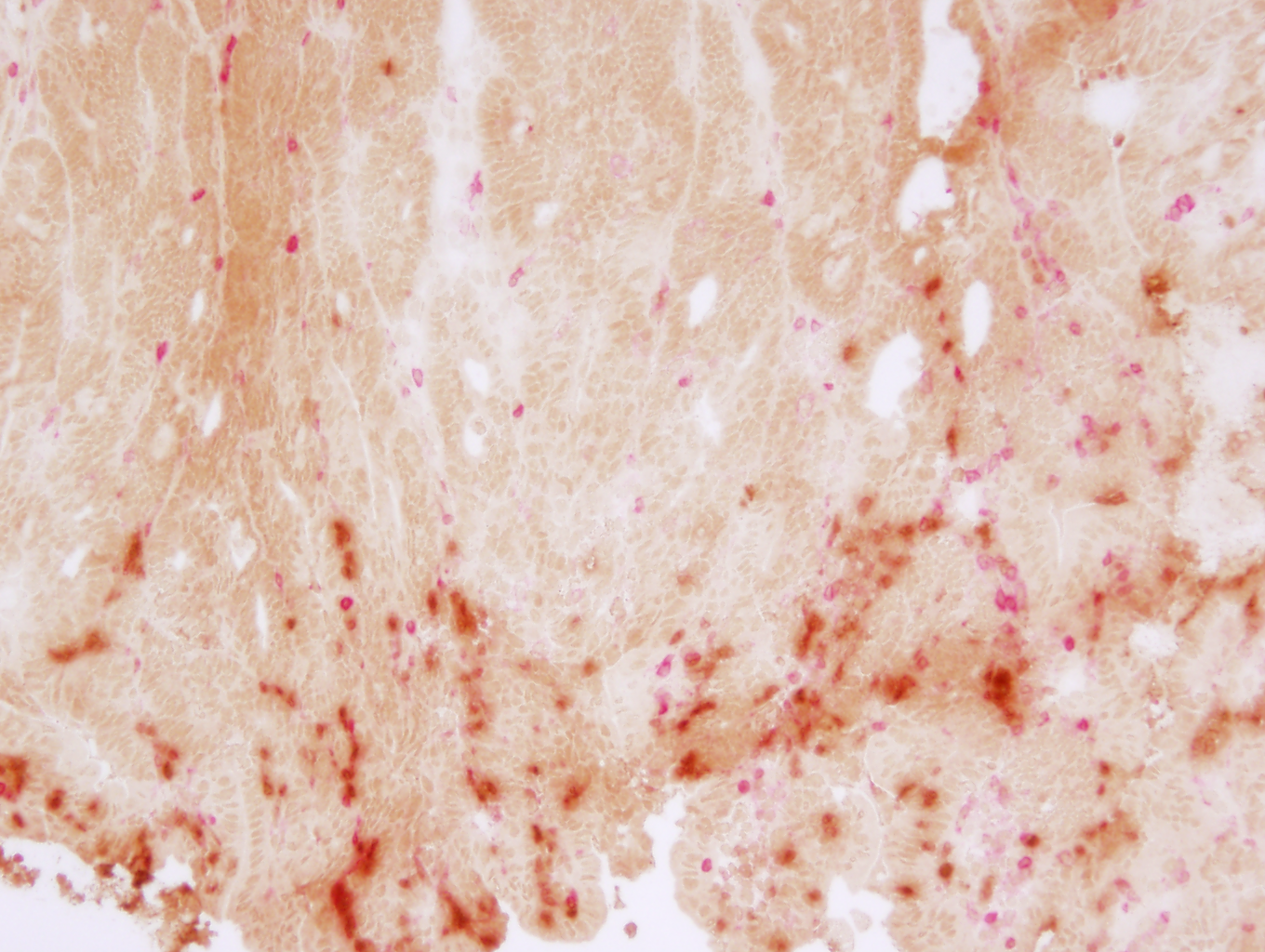

Supplement: Supplementary file 3 — Appendix Figure Source Data [file 44321_2024_100_MOESM3_ESM.zip › Appendix Figures/S7/CD3-Red MPO-Brown.tif]

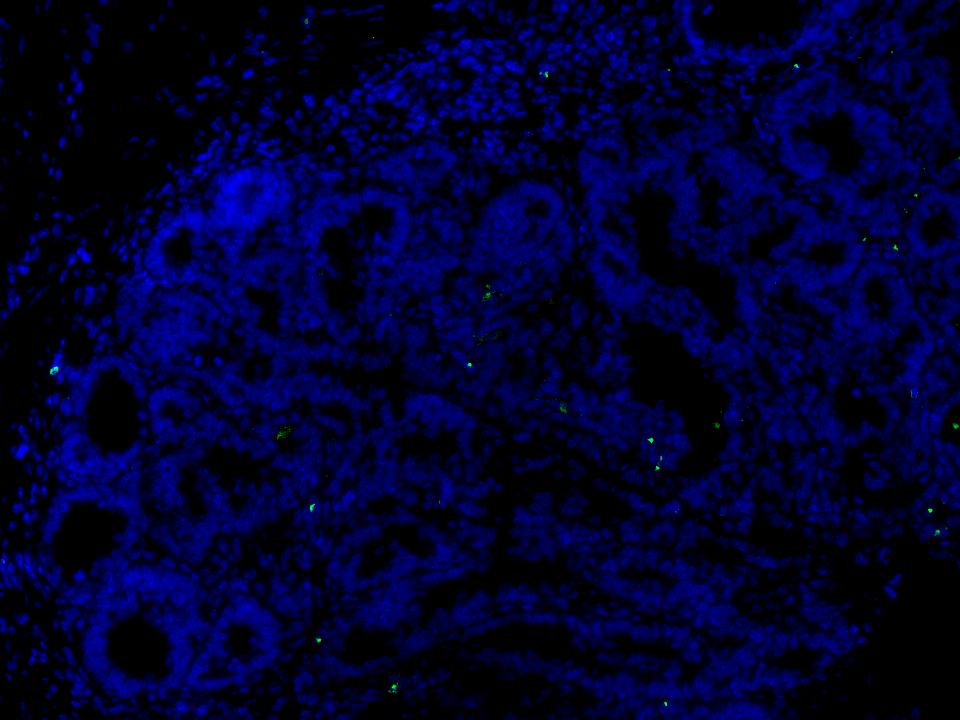

Supplement: Supplementary file 3 — Appendix Figure Source Data [file 44321_2024_100_MOESM3_ESM.zip › Appendix Figures/S7/Overlay_NK_DAPI.tif]

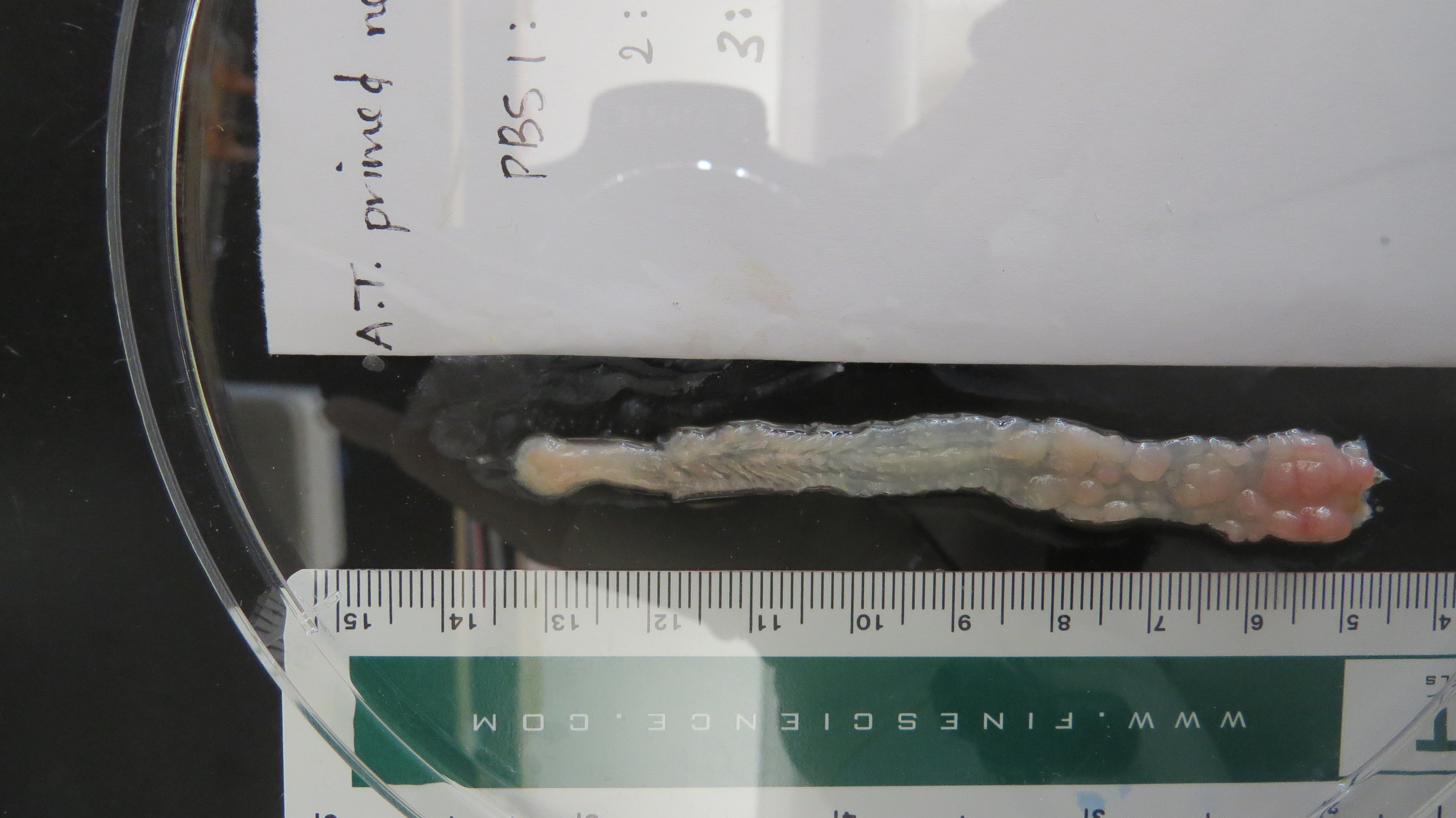

Supplement: Supplementary file 5 — Source data Fig. 2 [file 44321_2024_100_MOESM5_ESM.zip › Figure 2/2B/Figure 2B 1.JPG]

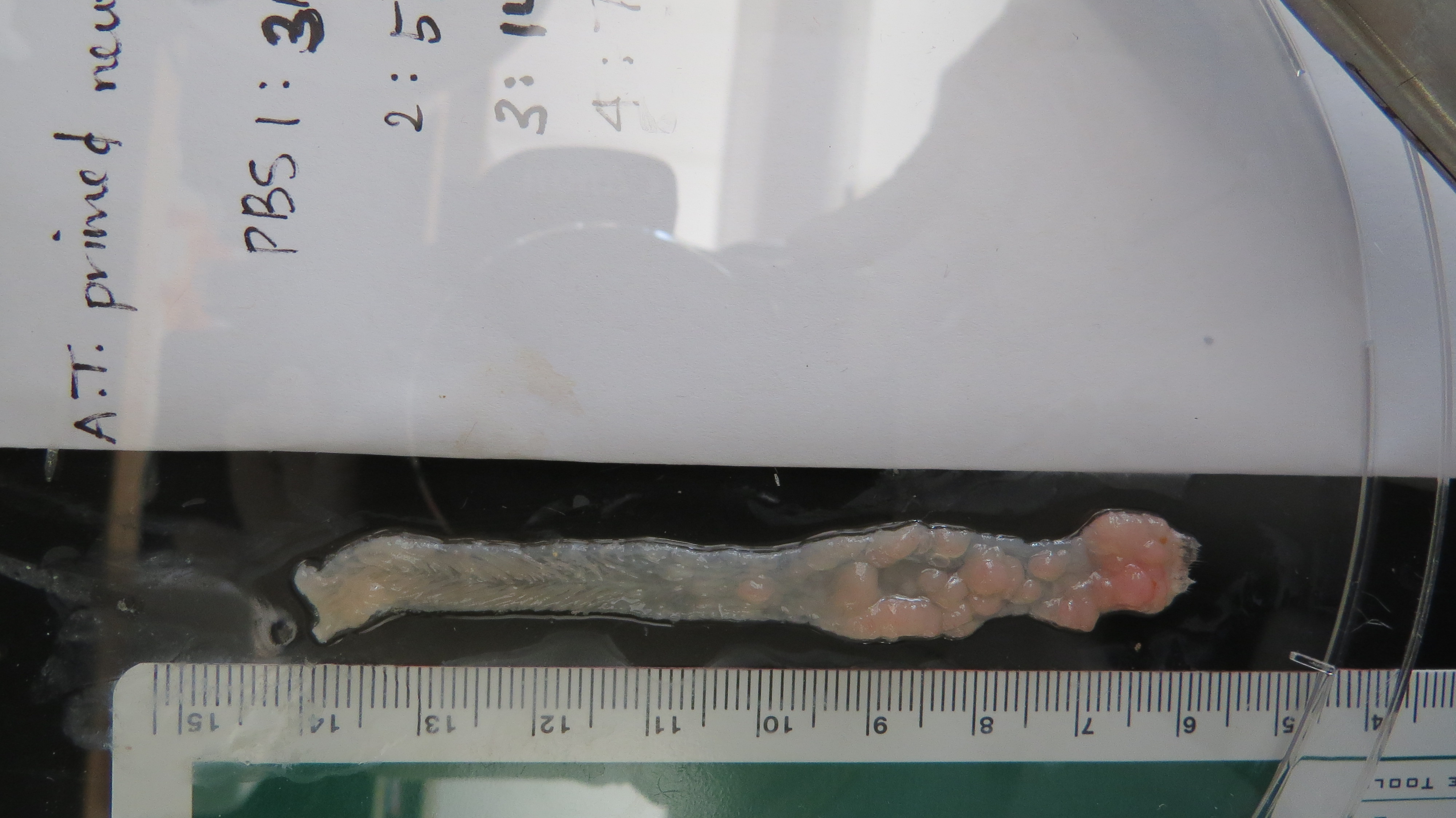

Supplement: Supplementary file 5 — Source data Fig. 2 [file 44321_2024_100_MOESM5_ESM.zip › Figure 2/2B/Figure 2B 2.JPG]

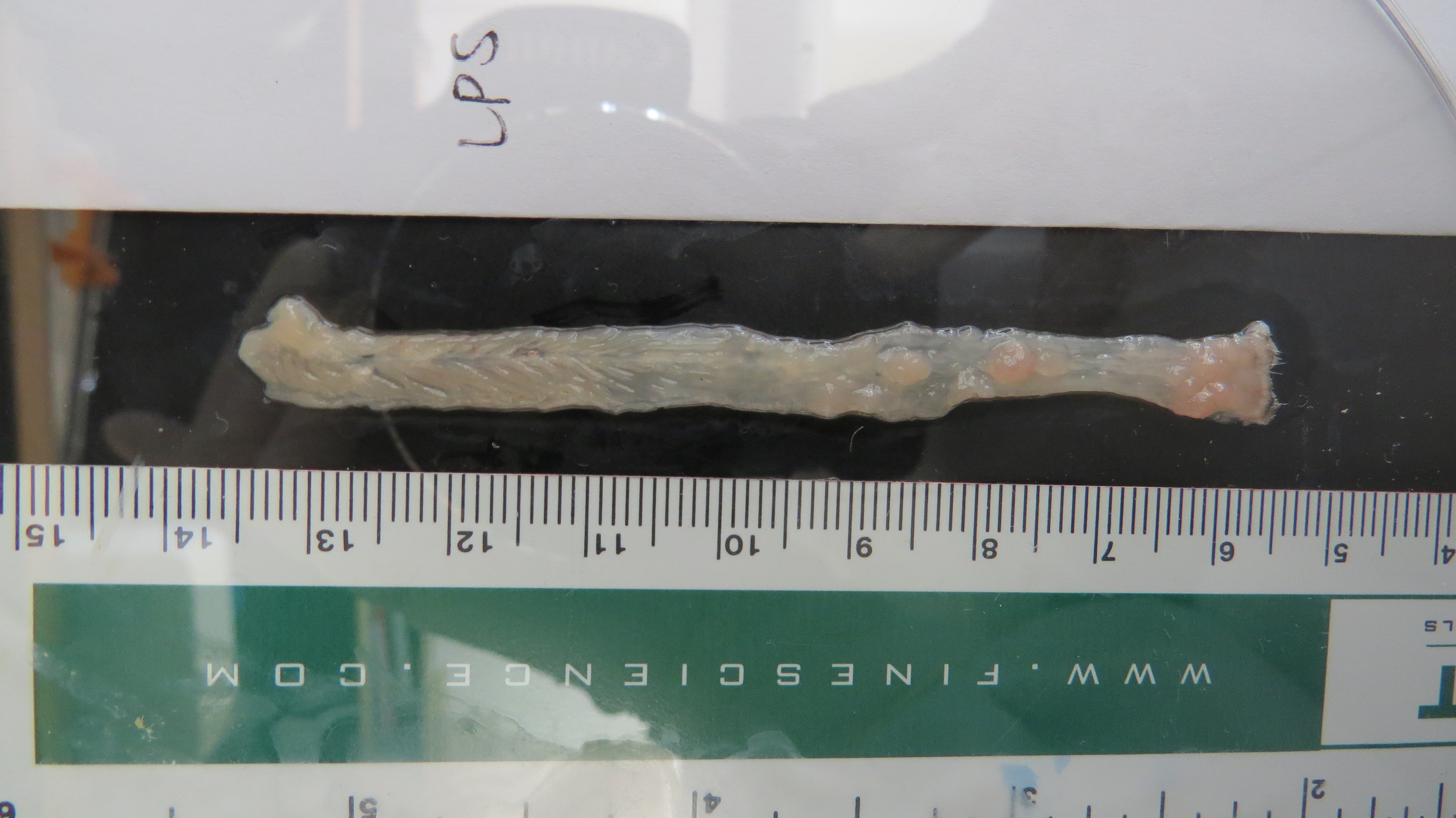

Supplement: Supplementary file 5 — Source data Fig. 2 [file 44321_2024_100_MOESM5_ESM.zip › Figure 2/2B/Figure 2B 3.JPG]

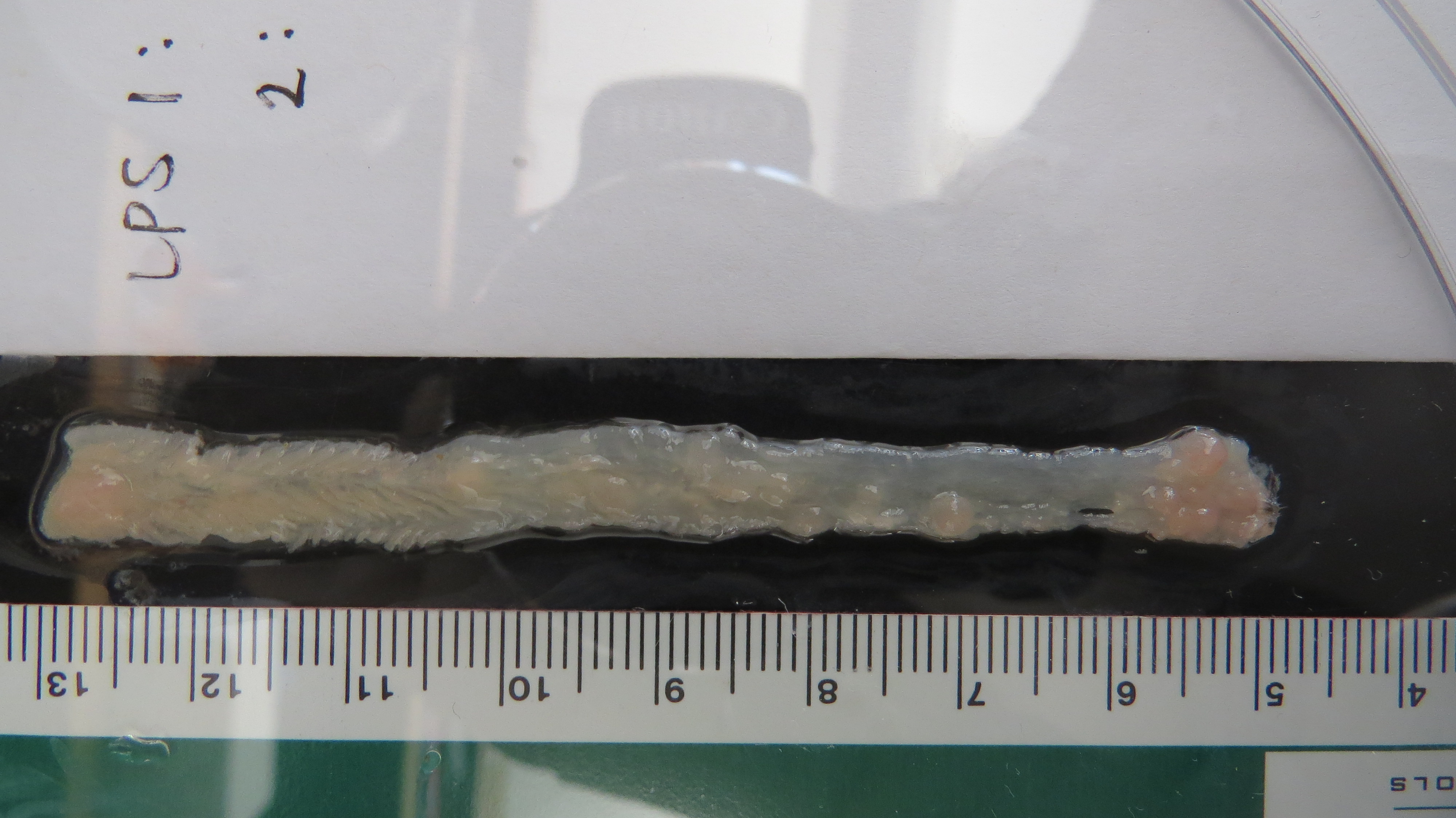

Supplement: Supplementary file 5 — Source data Fig. 2 [file 44321_2024_100_MOESM5_ESM.zip › Figure 2/2B/Figure 2B 4.JPG]

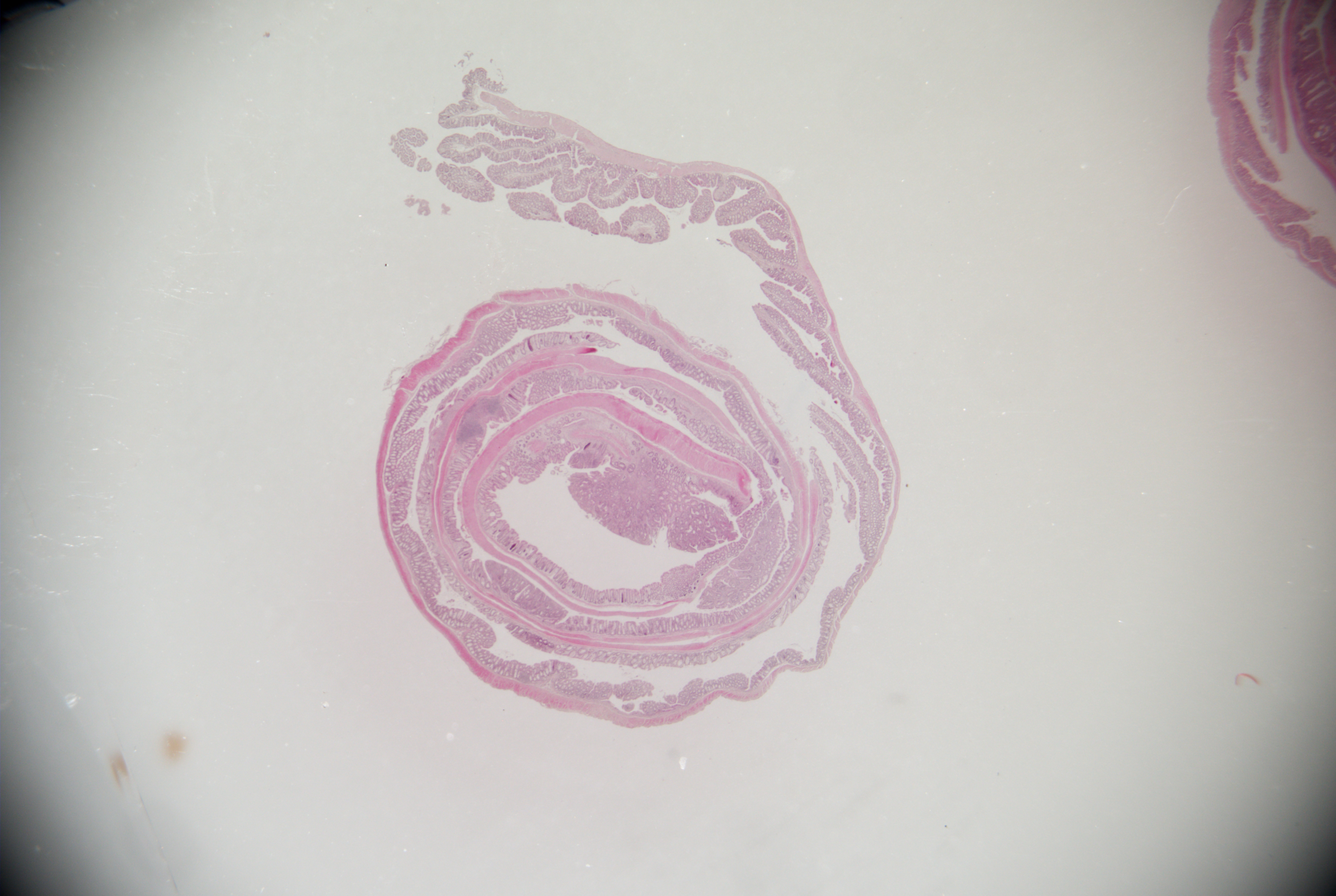

Supplement: Supplementary file 5 — Source data Fig. 2 [file 44321_2024_100_MOESM5_ESM.zip › Figure 2/2E/Figure 2E LPS.jpg]

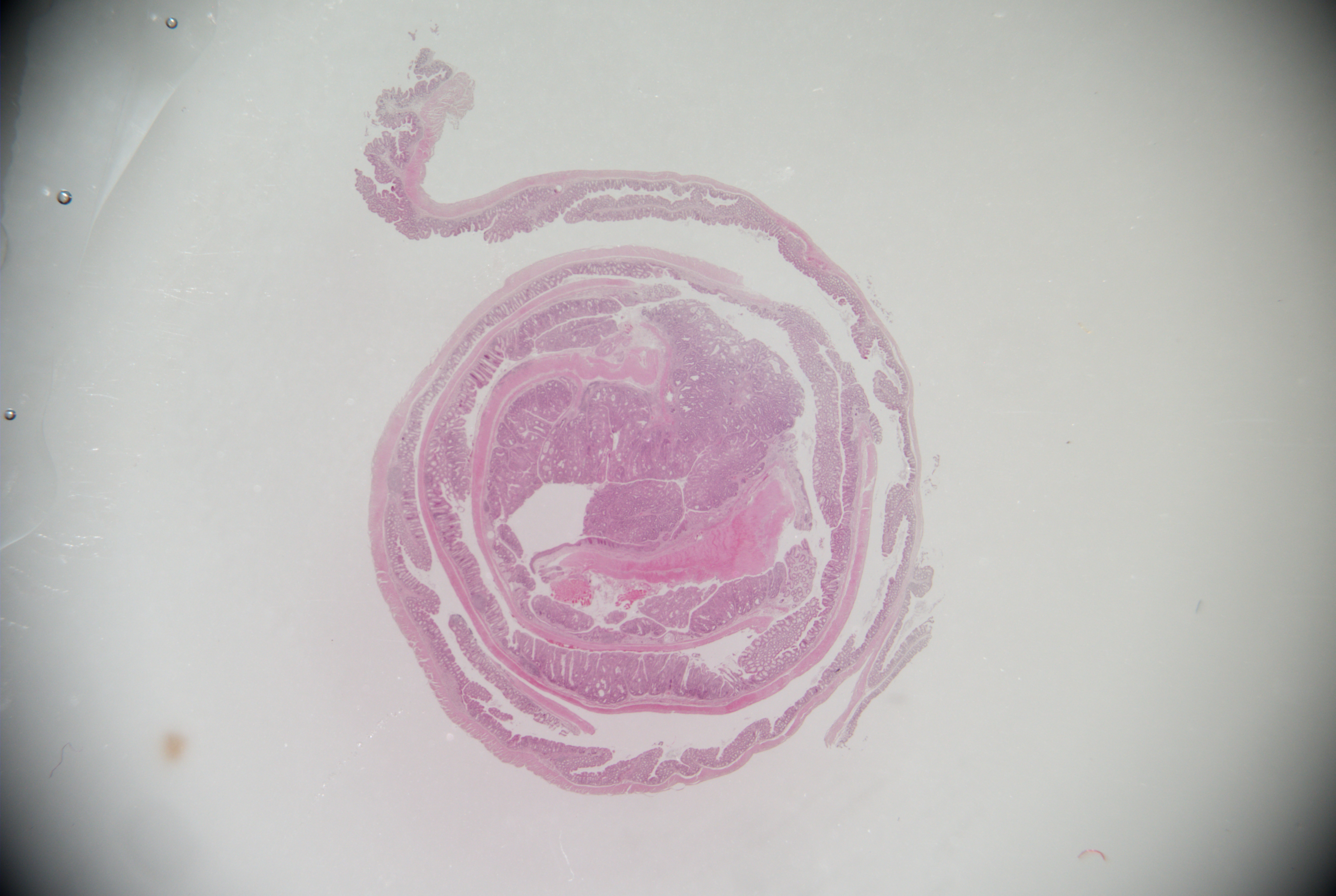

Supplement: Supplementary file 5 — Source data Fig. 2 [file 44321_2024_100_MOESM5_ESM.zip › Figure 2/2E/Figure 2E PBS.jpg]

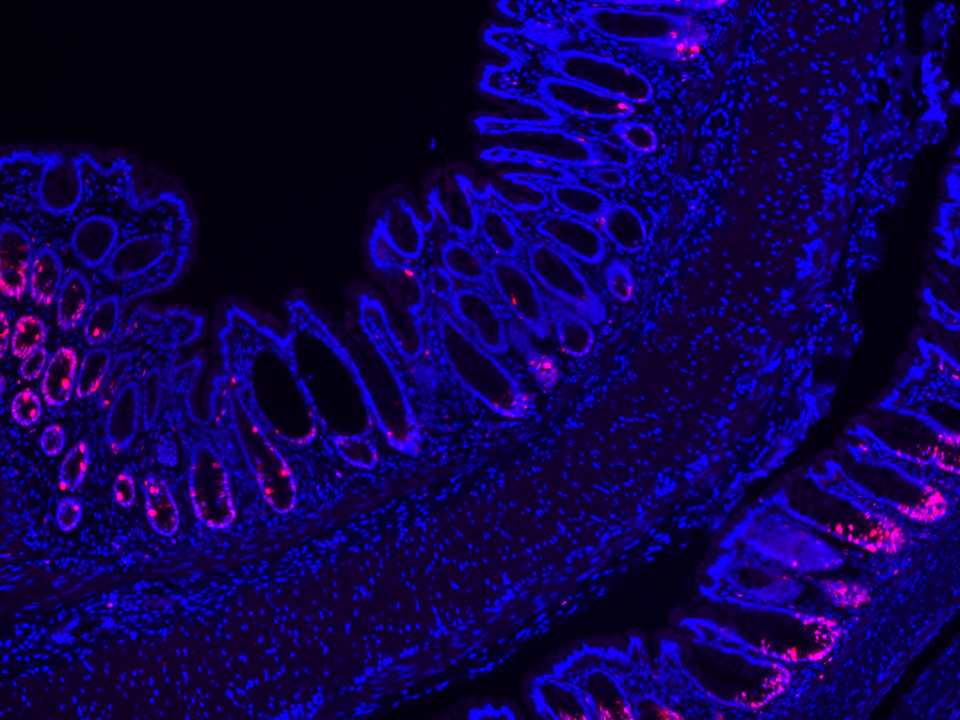

Supplement: Supplementary file 5 — Source data Fig. 2 [file 44321_2024_100_MOESM5_ESM.zip › Figure 2/2F/Figure 2F LPS.tif]

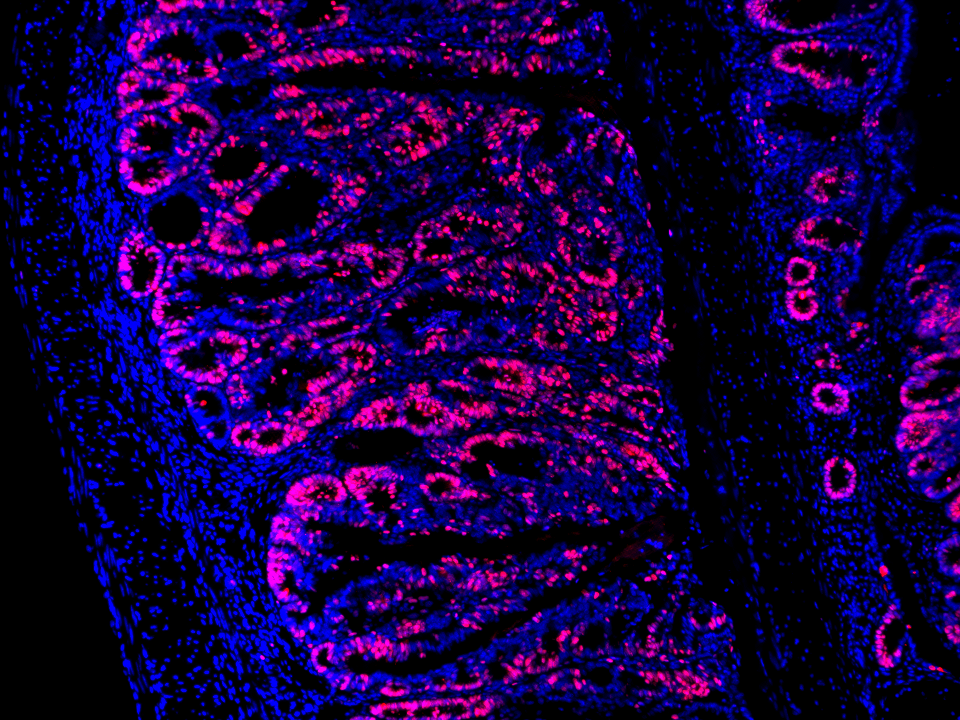

Supplement: Supplementary file 5 — Source data Fig. 2 [file 44321_2024_100_MOESM5_ESM.zip › Figure 2/2F/Figure 2F PBS.tif]

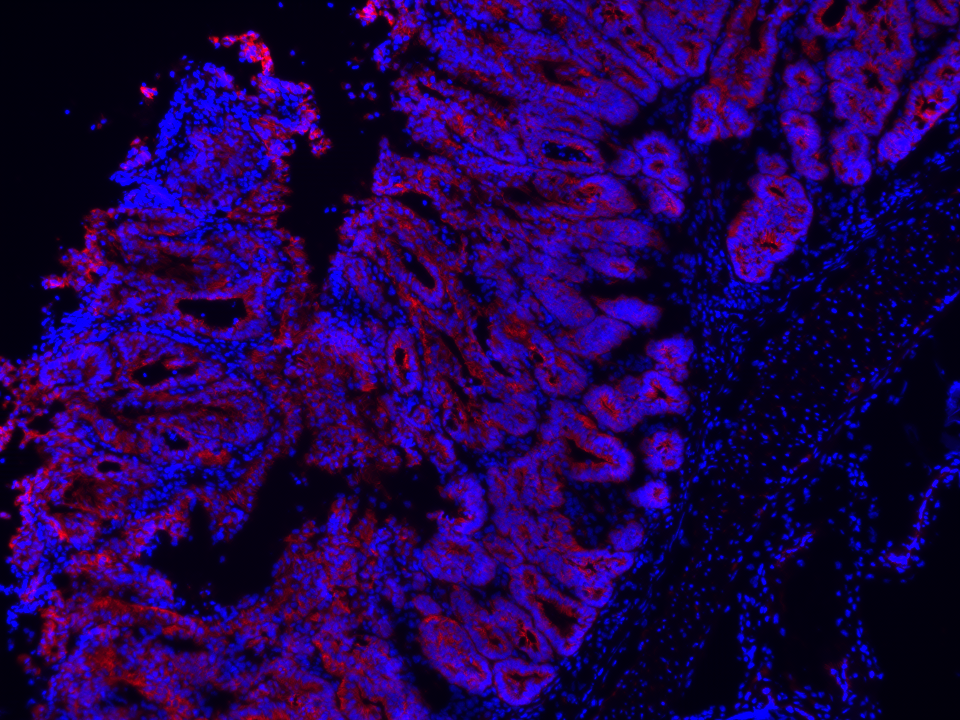

Supplement: Supplementary file 5 — Source data Fig. 2 [file 44321_2024_100_MOESM5_ESM.zip › Figure 2/2G/Figure 2G LPS.tif]

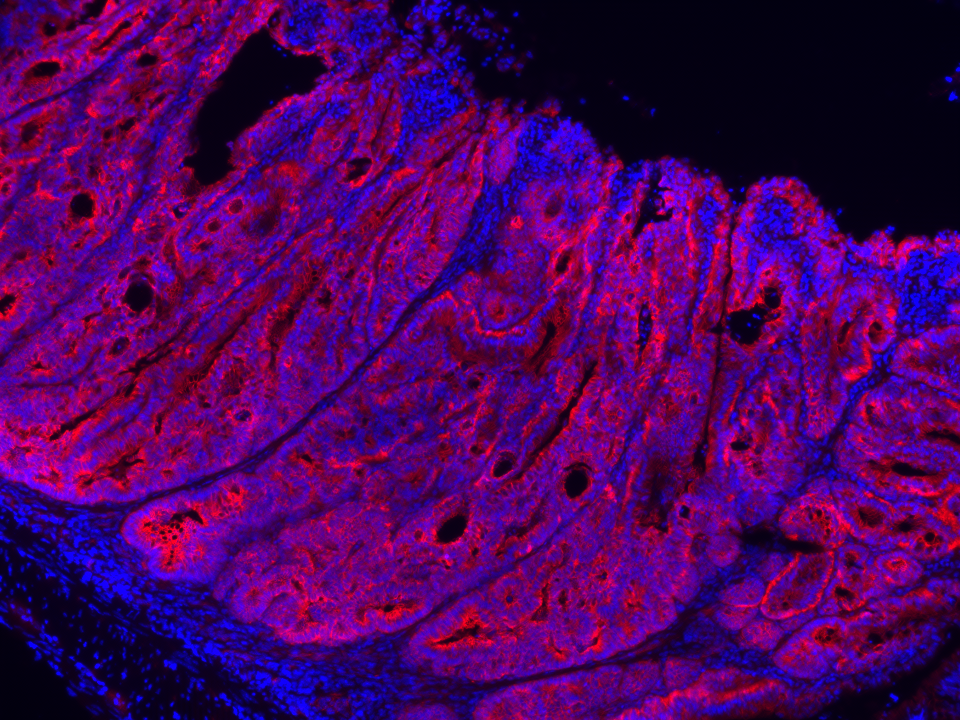

Supplement: Supplementary file 5 — Source data Fig. 2 [file 44321_2024_100_MOESM5_ESM.zip › Figure 2/2G/Figure 2G PBS.tif]

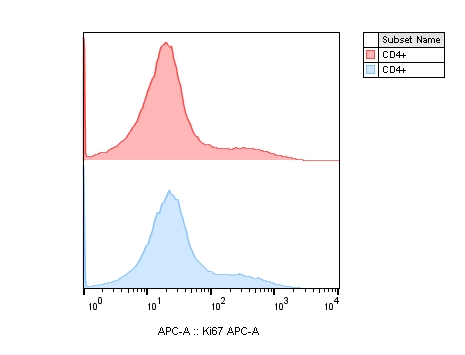

Supplement: Supplementary file 6 — Source data Fig. 3 [file 44321_2024_100_MOESM6_ESM.zip › Figure 3/3C/1.jpg]

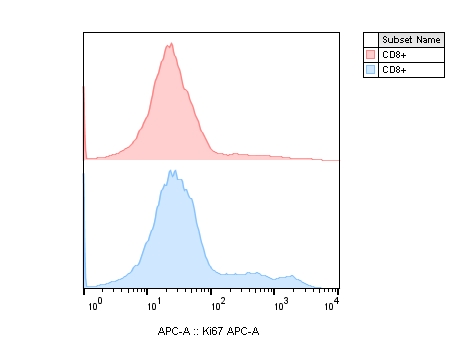

Supplement: Supplementary file 6 — Source data Fig. 3 [file 44321_2024_100_MOESM6_ESM.zip › Figure 3/3C/2.jpg]

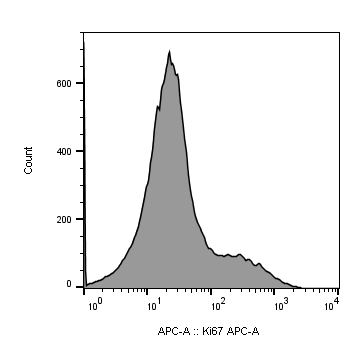

Supplement: Supplementary file 6 — Source data Fig. 3 [file 44321_2024_100_MOESM6_ESM.zip › Figure 3/3C/CD4 LPS.jpg]

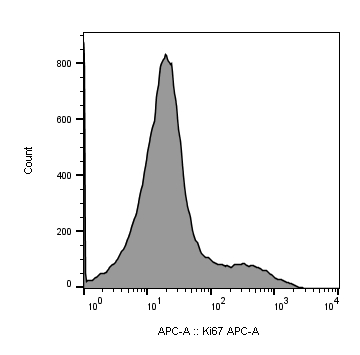

Supplement: Supplementary file 6 — Source data Fig. 3 [file 44321_2024_100_MOESM6_ESM.zip › Figure 3/3C/CD4 PBS.jpg]

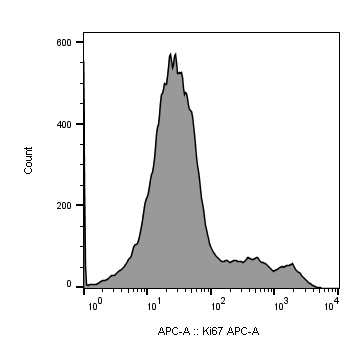

Supplement: Supplementary file 6 — Source data Fig. 3 [file 44321_2024_100_MOESM6_ESM.zip › Figure 3/3C/CD8 LPS.jpg]

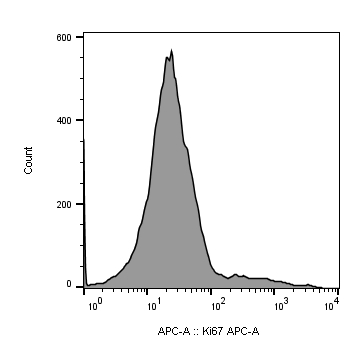

Supplement: Supplementary file 6 — Source data Fig. 3 [file 44321_2024_100_MOESM6_ESM.zip › Figure 3/3C/CD8 PBS.jpg]

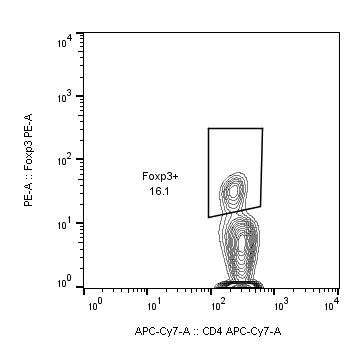

Supplement: Supplementary file 6 — Source data Fig. 3 [file 44321_2024_100_MOESM6_ESM.zip › Figure 3/3D/1.jpg]

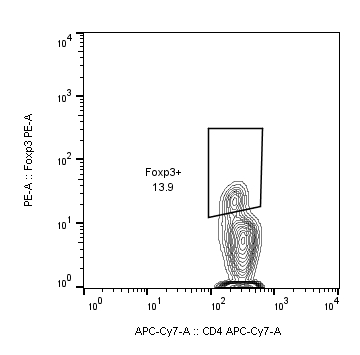

Supplement: Supplementary file 6 — Source data Fig. 3 [file 44321_2024_100_MOESM6_ESM.zip › Figure 3/3D/2.jpg]

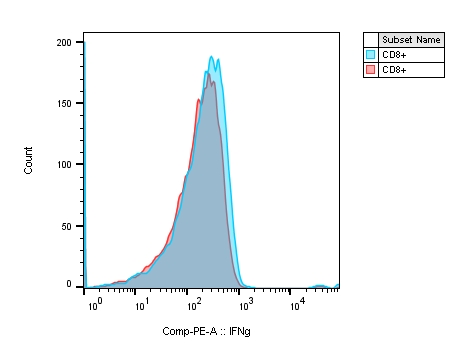

Supplement: Supplementary file 6 — Source data Fig. 3 [file 44321_2024_100_MOESM6_ESM.zip › Figure 3/3F/1.jpg]

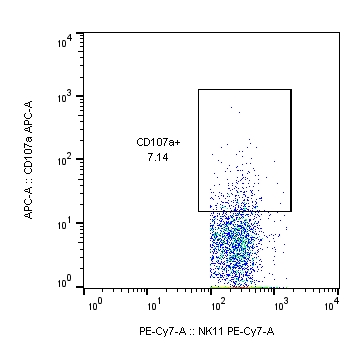

Supplement: Supplementary file 7 — Source data Fig. 4 [file 44321_2024_100_MOESM7_ESM.zip › Figure 4/4F/1.jpg]

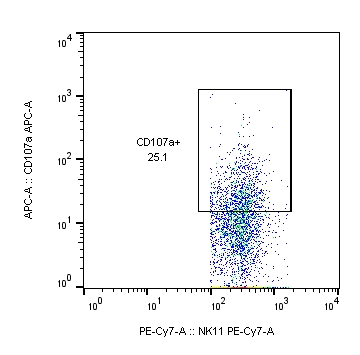

Supplement: Supplementary file 7 — Source data Fig. 4 [file 44321_2024_100_MOESM7_ESM.zip › Figure 4/4F/2.jpg]

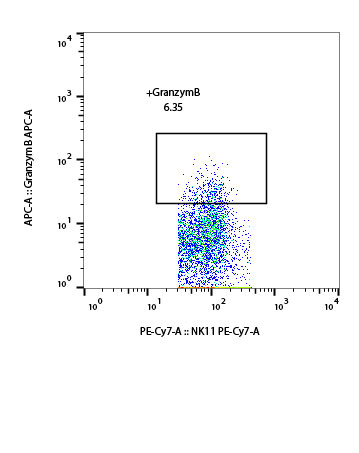

Supplement: Supplementary file 7 — Source data Fig. 4 [file 44321_2024_100_MOESM7_ESM.zip › Figure 4/4H/1.jpg]

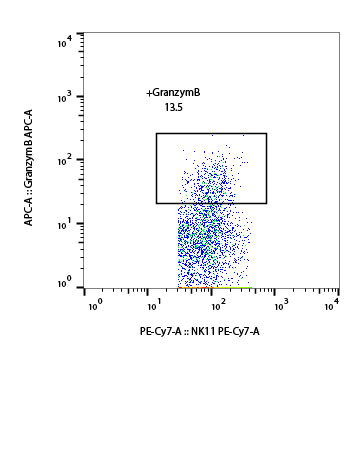

Supplement: Supplementary file 7 — Source data Fig. 4 [file 44321_2024_100_MOESM7_ESM.zip › Figure 4/4H/2.jpg]

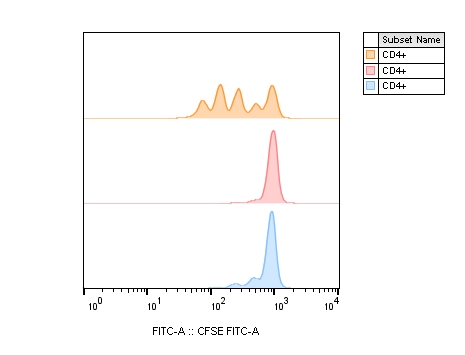

Supplement: Supplementary file 8 — Source data Fig. 5 [file 44321_2024_100_MOESM8_ESM.zip › Figure 5/5A/1.jpg]

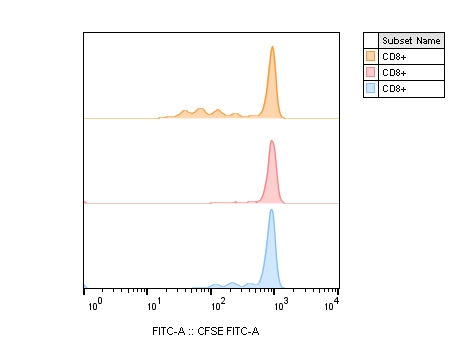

Supplement: Supplementary file 8 — Source data Fig. 5 [file 44321_2024_100_MOESM8_ESM.zip › Figure 5/5A/2.jpg]

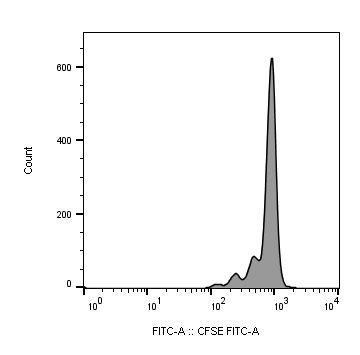

Supplement: Supplementary file 8 — Source data Fig. 5 [file 44321_2024_100_MOESM8_ESM.zip › Figure 5/5A/CD4 LPS.jpg]

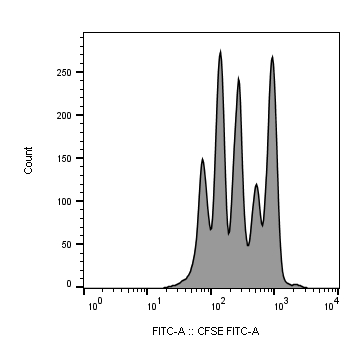

Supplement: Supplementary file 8 — Source data Fig. 5 [file 44321_2024_100_MOESM8_ESM.zip › Figure 5/5A/CD4 NoN.jpg]

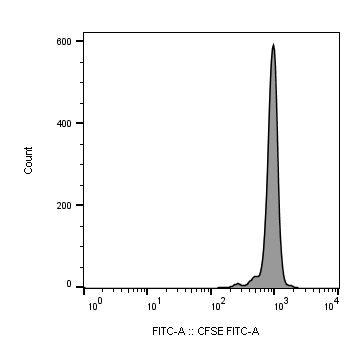

Supplement: Supplementary file 8 — Source data Fig. 5 [file 44321_2024_100_MOESM8_ESM.zip › Figure 5/5A/CD4 PBS.jpg]

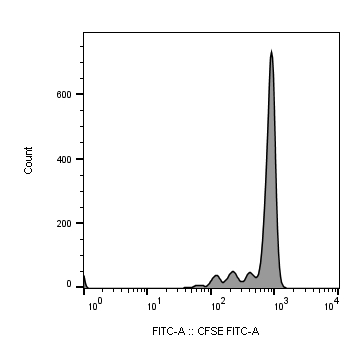

Supplement: Supplementary file 8 — Source data Fig. 5 [file 44321_2024_100_MOESM8_ESM.zip › Figure 5/5A/CD8 LPS.jpg]

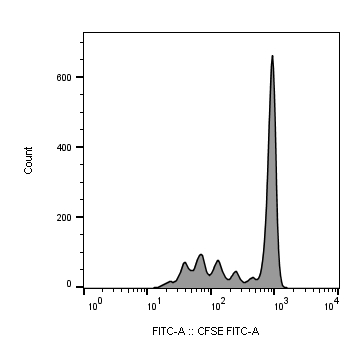

Supplement: Supplementary file 8 — Source data Fig. 5 [file 44321_2024_100_MOESM8_ESM.zip › Figure 5/5A/CD8 NoN.jpg]

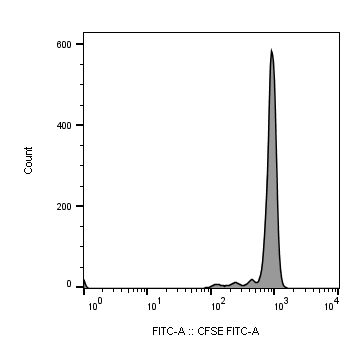

Supplement: Supplementary file 8 — Source data Fig. 5 [file 44321_2024_100_MOESM8_ESM.zip › Figure 5/5A/CD8 PBS.jpg]

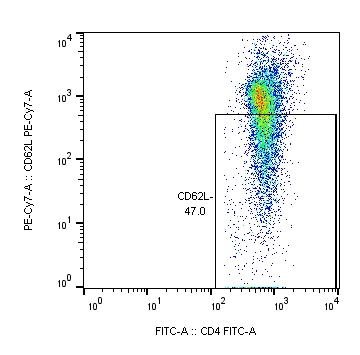

Supplement: Supplementary file 8 — Source data Fig. 5 [file 44321_2024_100_MOESM8_ESM.zip › Figure 5/5C/CD4 LPS.jpg]

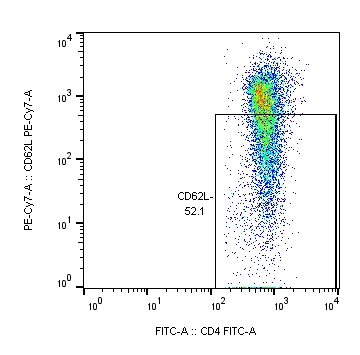

Supplement: Supplementary file 8 — Source data Fig. 5 [file 44321_2024_100_MOESM8_ESM.zip › Figure 5/5C/CD4 NoN.jpg]

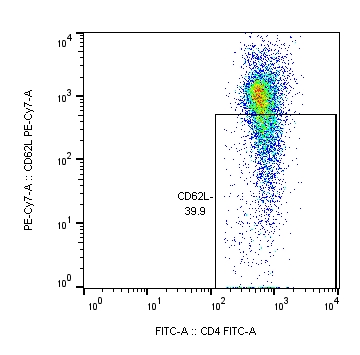

Supplement: Supplementary file 8 — Source data Fig. 5 [file 44321_2024_100_MOESM8_ESM.zip › Figure 5/5C/CD4 PBS.jpg]

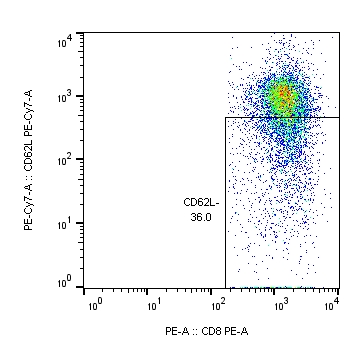

Supplement: Supplementary file 8 — Source data Fig. 5 [file 44321_2024_100_MOESM8_ESM.zip › Figure 5/5C/CD8 LPS.jpg]

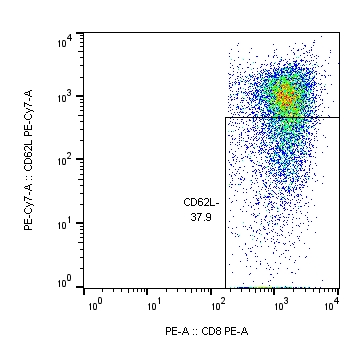

Supplement: Supplementary file 8 — Source data Fig. 5 [file 44321_2024_100_MOESM8_ESM.zip › Figure 5/5C/CD8 NoN.jpg]

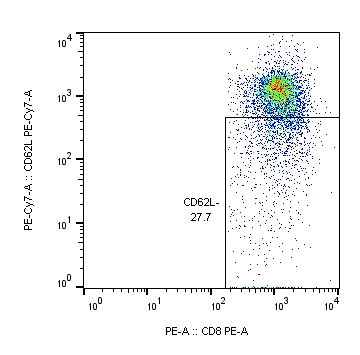

Supplement: Supplementary file 8 — Source data Fig. 5 [file 44321_2024_100_MOESM8_ESM.zip › Figure 5/5C/CD8 PBS.jpg]

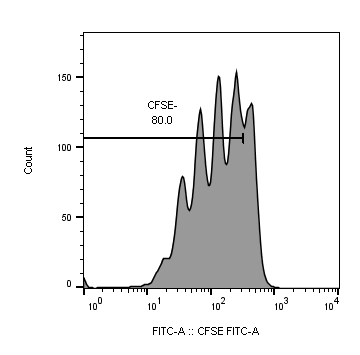

Supplement: Supplementary file 9 — Source data Fig. 6 [file 44321_2024_100_MOESM9_ESM.zip › Figure 6/6A/1.jpg]

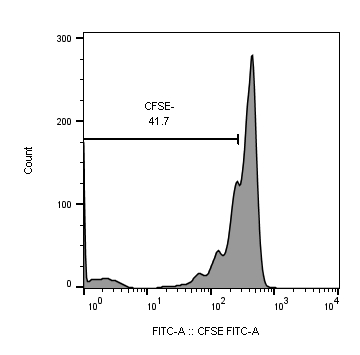

Supplement: Supplementary file 9 — Source data Fig. 6 [file 44321_2024_100_MOESM9_ESM.zip › Figure 6/6A/2.jpg]

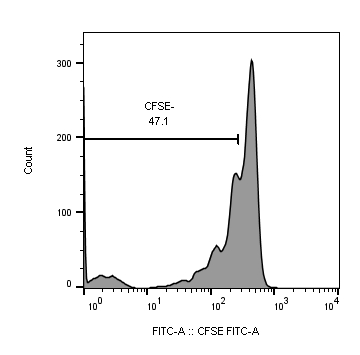

Supplement: Supplementary file 9 — Source data Fig. 6 [file 44321_2024_100_MOESM9_ESM.zip › Figure 6/6A/3.jpg]

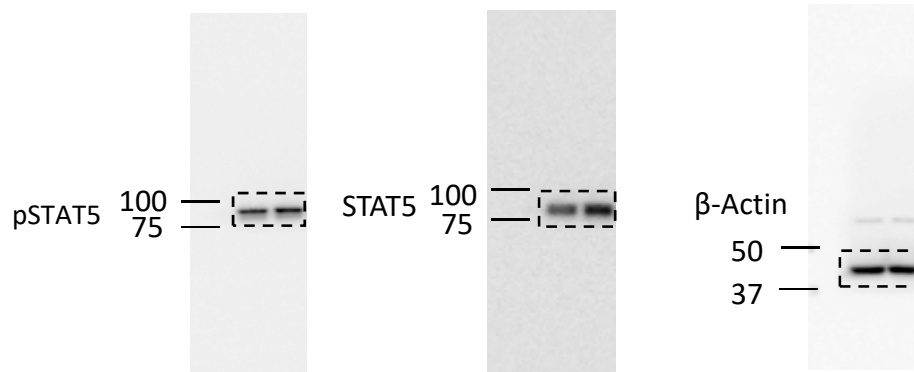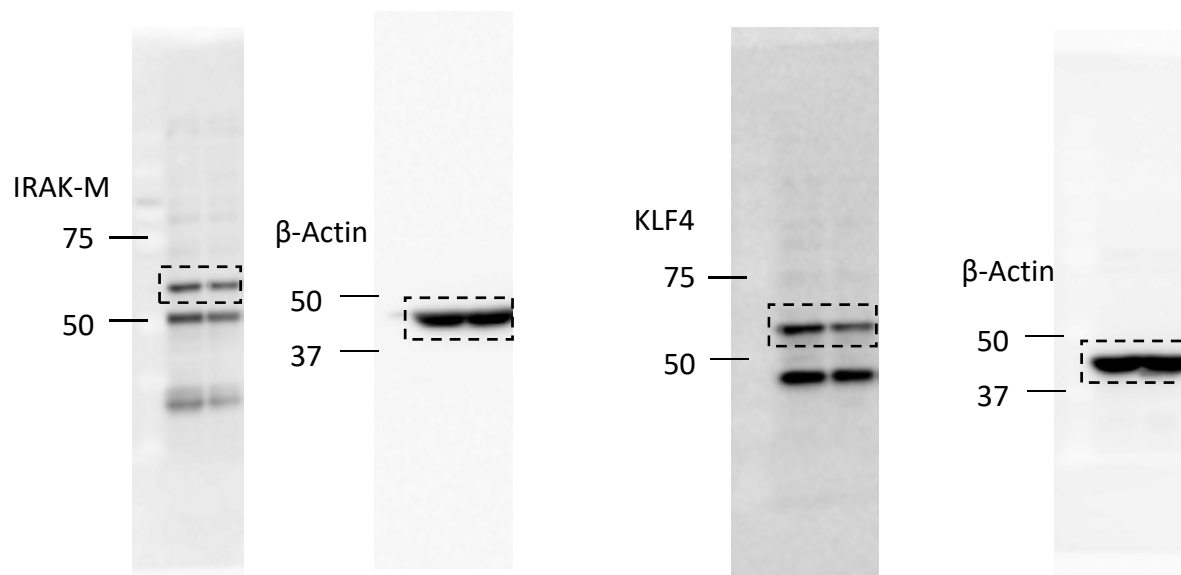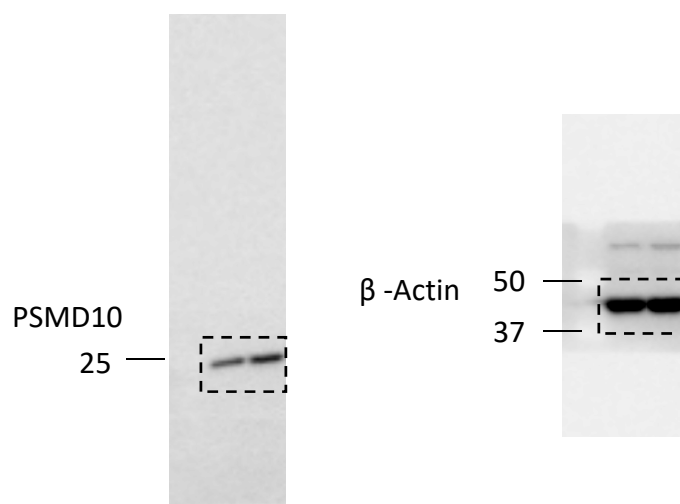

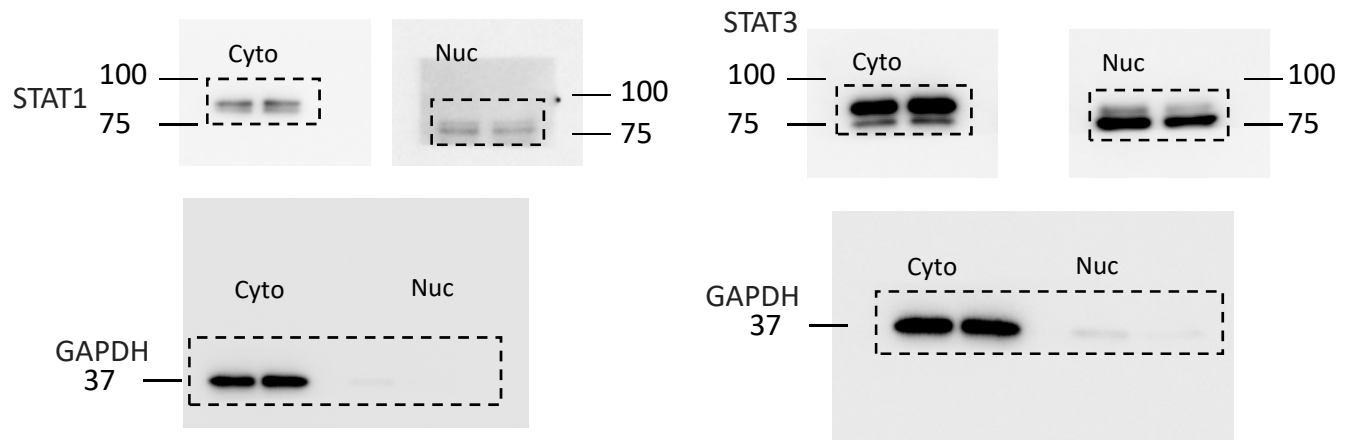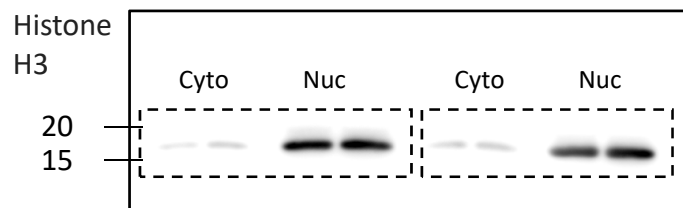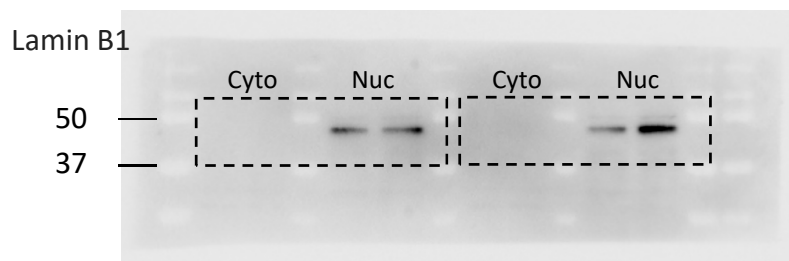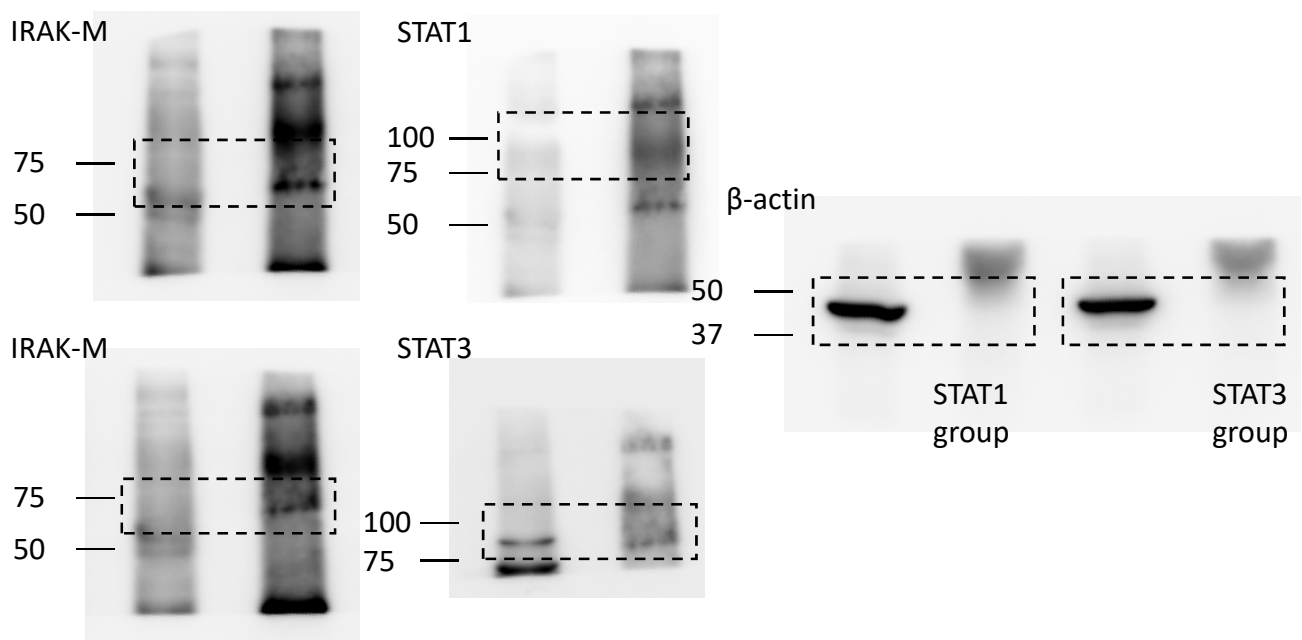

Supplement: Supplementary file 10 — Source data Fig. 7 [file 44321_2024_100_MOESM10_ESM.zip › Figure 7/Gel original.pdf]
